# Presurgical Functional Cortical Mapping Using Electromagnetic Source Imaging

**DOI:** 10.3389/fneur.2019.00628

**Published:** 2019-06-13

**Authors:** Rudolf Kreidenhuber, Xavier De Tiège, Stefan Rampp

**Affiliations:** ^1^Department of Neurology, Christian-Doppler Medical Center, Paracelsus Medical University, Salzburg, Austria; ^2^Centre for Cognitive Neuroscience, University of Salzburg, Salzburg, Austria; ^3^Laboratoire de Cartographie Fonctionelle du Cerveau, ULB Neuroscience Institute, Université Libre de Bruxelles, Brussels, Belgium; ^4^Department of Functional Neuroimaging, Service of Nuclear Medicine, CUB Hôpital Erasme, Université Libre de Bruxelles, Brussels, Belgium; ^5^Department of Neurosurgery, University Hospital Erlangen, Erlangen, Germany; ^6^Department of Neurosurgery, University Hospital Halle, Halle, Germany

**Keywords:** magnetoencephalography, functional cortical mapping, fMRI, EEG, presurgical

## Abstract

Preoperative localization of functionally eloquent cortex (functional cortical mapping) is common clinical practice in order to avoid or reduce postoperative morbidity. This review aims at providing a general overview of magnetoencephalography (MEG) and high-density electroencephalography (hdEEG) based methods and their clinical role as compared to common alternatives for functional cortical mapping of (1) verbal language function, (2) sensorimotor cortex, (3) memory, (4) visual, and (5) auditory cortex. We highlight strengths, weaknesses and limitations of these functional cortical mapping modalities based on findings in the recent literature. We also compare their performance relative to other non-invasive functional cortical mapping methods, such as functional Magnetic Resonance Imaging (fMRI), Transcranial Magnetic Stimulation (TMS), and to invasive methods like the intracarotid Amobarbital Test (WADA-Test) or intracranial investigations.

## Introduction

Functional cortical mapping (FCM) aims at localizing eloquent functional cortex using a range of invasive and non-invasive methods (1). Its main indication is to characterize the anatomical relationship between functionally eloquent cortex and the extent of a planned surgical resection, e.g., of an intracranial tumor or the putative epileptogenic zone in patients with pharmacoresistant focal epilepsy. In the latter patient group, results of FCM are usually interpreted in conjunction with structural magnetic resonance imaging (MRI), neuropsychological findings, positron emission tomography (PET), single photon emission computed tomography (SPECT) and video-electroencephalography (EEG) monitoring (1).

The advent of non-invasive FCM methods has substantially influenced the care of neurosurgical candidates. Indeed, the availability of FCM results based on non-invasive approaches before surgery allows for a better estimation of the risk-benefit ratio of the planned neurosurgical procedure with better patients' counsel, optimized neurosurgical strategies, as well as tailored resection extents. Overlap between functionally eloquent cortex as identified by non-invasive FCM and lesional or epileptogenic zones may even argue against surgery or for alternative therapeutic strategies.

This paper reviews currently available FCM methods with a special emphasis on electromagnetic source imaging. Strengths, weaknesses and limitations of electromagnetic source imaging in relation to other modalities commonly used for mapping of verbal language, sensorimotor, memory, visual, and auditory functions are also presented.

### Functional Cortical Mapping Methods

#### Non-invasive Methods

##### Anatomical Landmarks

Identification of anatomical landmarks in structural cerebral imaging represents an easy and fast approach to localize functionally eloquent cortex. However, inter-rater reliability is significantly lower than with FCM results, even within the same subject and across successive analyses over several days (2). Furthermore, anatomic variability, lesion-induced plasticity, and displacement considerably limit accuracy and viability of this approach [for references, see, e.g., (3, 4)].

##### Functional Magnetic Resonance Imaging (fMRI)

Among the various non-invasive FCM techniques that can be used in humans, fMRI is by far the most commonly used (5). Active brain areas are detected indirectly by relying mostly on task-related changes in regional brain perfusion. Blood oxygenation level dependent (BOLD) signal changes can then be detected by fMRI. The resulting spatial resolution of fMRI is excellent (~1 mm, including deep locations). However, the dependence on the comparably slow hemodynamic response limits its temporal resolution (~1 s) (6). Furthermore, the neurovascular coupling may be altered by lesional processes in the vicinity, potentially leading to spurious fMRI results (7, 8). Moreover, activation patterns of single patients with brain disorders can be more difficult to interpret than those obtained in individuals or groups of healthy subjects (8, 9).

##### Electric Source Imaging (ESI) and Magnetic Source Imaging (MSI)

Magnetoencephalography (MEG) and EEG are non-invasive methods that record magnetic and electric fields, respectively. These are generated by excitatory or inhibitory postsynaptic potentials at the apical dendrites of neocortical pyramidal cells. Determination of the anatomical location of the sources generating the measured signal is known as magnetic and electric source imaging (MSI and ESI, respectively). This is achieved by combining MEG or EEG data with structural MRI. For the purpose of clarity, we will henceforth only use MEG and EEG to refer to MSI- or ESI-based FCM.

In contrast to EEG, MEG is sensitive mainly to tangential neocortical source components. Consequently, it detects activity mainly from the sulcal walls, while small areas at the crown or in the sulcal depth barely contribute to detectable signals (10). Sensitivity of both EEG and MEG decreases with increasing cortical depth. The number of sensors, amplitude of background activity and, in EEG, smearing of the field distribution due to variations in skull resistivity, account for differences in recorded signals (11). For a comprehensive review on mechanisms of MEG and EEG signal generation, see (12). Data from EEG and MEG are complementary and combined analysis has been shown to outperform the single modalities alone in the presurgical evaluation of patients with epilepsy in the context of source localization (13). Still, this combined approach is rarely used for FCM and comparable studies for simultaneous FCM in MEG and EEG are, to the best of our knowledge, lacking.

##### Transcranial Magnetic Stimulation (TMS)

TMS is a non-invasive form of neurostimulation. Magnetic fields applied focally are used to induce or inhibit electric activity of targeted neurons via electromagnetic induction. Neuronavigation using the patient's individual structural MRI (and eventually results from other FCM modalities) allows for better targeted stimulation (nTMS). In the context of FCM, it is used primarily for verbal language and primary motor (M1) cortex localization (14–17) in specialized centers.

#### Invasive Methods

##### Intracarotid Amobarbital Test (IAT)

Wada and Rasmussen described in 1960 the intracarotid injection of amobarbital for the lateralization of cerebral speech dominance (18). Sodium amobarbital is injected into a single internal carotid artery via transfemoral arterial catheterization. This procedure transiently suppresses neuronal function of the corresponding brain hemisphere depending on the respective vascular supply, mainly the ipsilateral anterior and middle cerebral arteries. Patients then undergo verbal language and neuropsychological testing during the transient unilateral hemispheric anesthesia, evaluating language and memory functions. The procedure is then repeated for the contralateral hemisphere. The IAT or “Wada-Test” allows for lateralization but not localization of brain areas involved in verbal language and memory functions (19). Limitations and shortcomings include risks of stroke, hemorrhage, infection (morbidity around 3–5%) and the possibility of arterial crossflow to the contralateral hemisphere via the circle of Willis—for reviews or discussions, see, e.g., (20, 21).

##### Direct Current Stimulation

Direct current stimulation (DCS) is used in awake craniotomy for language-, motor- and memory mapping. For a review, see (22). Intracranial EEG electrodes record local field potentials and can also be used for stimulation purposes. Cortex areas are labeled as eloquent either via gain/loss of the specific function, i.e., motor jerks, speech arrest, memory deficits, or via alterations of simultaneously recorded local field potentials or targeted electromyography (22).

## EEG/MEG Functional Cortical Mapping Compared to Other Methods

### Verbal Language Function

Presurgical and potentially intraoperative investigation of verbal language function is mandatory to avoid postoperative language deficits if surgery involves resection near (presumed) language eloquent cortex.

Language related cortex is extensive and bilateral, although one hemisphere is usually dominant. Two broad processing streams can be distinguished: a ventral stream for speech comprehension (bilateral, temporal lobes) and a dorsal stream for sensory-motor integration (asymmetric, temporo-parietal junction, and frontal lobe). For comprehensive reviews, see, e.g., (23, 24). In a healthy population, ~95% of right-handers and ~76% of left-handers have left—hemisphere language dominance (25, 26). In patients with epilepsy, the rate of left—hemisphere language dominance drops significantly, with rates of 63−96% (right-handers) and 48−75% (left-handers) (26, 27).

Assessment of hemispheric dominance and intra-hemispheric cortical representation of speech processing and production are the main objectives of language FCM (5). IAT has been considered the gold standard for the evaluation of language hemispheric dominance. However, this role has been challenged by non-invasive methods (21). fMRI is the most widely used modality for assessment of language function, but EEG/MEG allow for characterization of temporal, spectral and also spatial dynamics of receptive and expressive language processing (5, 28).

#### Assessment of Hemispheric Dominance of Receptive Verbal Language Function

A frequently used approach for assessment of hemispheric receptive verbal language dominance was proposed by Papanicolaou et al. (29). It evaluates late (about 200 and 800 ms post stimulus) event related fields by use of an equivalent current dipole model. This approach showed consistent concordance of 86–92% between MEG and IAT results (29–33) and with findings from intracranial cortical stimulation (34).

Other strategies apply beamforming (35) and evaluate the spatial distribution of oscillatory changes related to silent reading. Lateralization of desynchronized/suppressed beta- (13–25 Hz) and gamma-activity (25–50 Hz) in regions of interest is analyzed and used to calculate a laterality index. This method showed concordance with IAT results in 95% of patients. Wilenius et al. (36) found a sensitivity of 67% and a specificity of 100% of stronger MEG responses to vowels than tones in the left hemisphere.

Furthermore, distributed source models, such as e.g., MR-FOCUSS have been successfully used for verbal language lateralization, as demonstrated by Bowyer et al. (37). Their approach of laterality index determination for multiple time intervals showed agreement with IAT results in 89%.

Active participation for the assessment of verbal language dominance using MEG may not be needed, as passive (listening) paradigms have also been described (38, 39).

#### Intrahemispheric Representation of Speech Processing and Language Production

IAT does not allow for an arterial injection of amobarbital that is selective enough to discriminate the sublobar structures involved in verbal language processing. Therefore, in clinical practice, intrahemispheric verbal language localization is primarily evaluated using fMRI or MEG preoperatively, or using direct current stimulation (DCS) intraoperatively.

Various protocols for the assessment of areas involved in verbal language comprehension (29, 40–42) and production (43, 44) are in routine clinical and research use. Using fMRI or MEG results as starting and end points for tractography further allows estimation of functional white matter pathways for planning of surgery or targeted intraopearative DCS testing (45).

Most studies suggest a high inter-subject variability of language-related activations, as well as high degrees of cortical plasticity in patients with brain disorders (28, 46, 47). Even DCS results have been shown to provide an incomplete representation of verbal language function with consecutive postoperative functional deficits (48).

Furthermore, nTMS, as well as nTMS-based DTI fiber tracking, have been applied successfully (49) but are, to the best of our knowledge, not currently in widespread clinical use.

#### Validation and Comparison

fMRI possibly provides better prediction of postoperative verbal language and memory deficits compared to IAT (50, 51), and shows concordance with IAT results in about 80–90% of cases (8). fMRI holds the potential to replace IAT for determination of hemispheric language dominance in many cases (5, 52). However, sites of fMRI activation do not necessarily reflect cortex essential for verbal language function and conversely, areas not activated by the fMRI paradigm under use may prove to be relevant (5). The precise intra-hemispheric localization of essential verbal language areas, especially in patients, remains suboptimal (5, 7, 8). Sensitivity of fMRI for DCS sites ranges from 59 to 100%, specificity from 0 to 97% (53).

Multiple approaches have been evaluated and validated for MEG verbal language lateralization. Stimulation paradigms usually test for language comprehension or language production. Agreement with IAT is about 86–92% for word recognition tasks (31, 32), and 78–82% for language production, depending on the specific experimental design (28).

Data on the comparison of localization accuracy of MEG mapping and DCS are sparse (54). Hirata et al. (42) reported distances between MEG activation maxima and DCS positive sites of 6.0 ± 7.1 mm, Simos et al. (34) described concordant results in a case report. Babajani-Feremi et al. (55) showed that the combination of fMRI, high-gamma electrocorticography (ECoG) and MEG predicted postoperative language decline best, while integrating fMRI and MEG provided the best trade-off between model complexity and prediction accuracy. Tarapore et al. (56) compared TMS, DCS, and MEG for language mapping in the same patients and found high sensitivity and specificity (90 and 98%) of TMS for DCS results in a population of 12 patients with lesions around cortical language areas, while MEG results correlated with TMS sites only in 5 subjects and DCS sites in 2 subjects. Other studies support the high sensitivity of TMS for DCS positive sites, but find reduced specificity, e.g., 90.2 and 23.8% by Picht et al. (57). Ille et al. (58) report a sensitivity of 100% and a specificity of 8%. In a separate study (59), the same group could achieve a sensitivity of 98% and a specificity of 83% by combining the results of TMS and fMRI. Comparative EEG, DCS, and/or invasive EEG data for language mapping is not available.

Clinical MEG societies regard MEG as a validated tool for presurgical evaluation of patients in respect to the assessment of verbal language-dominant hemisphere and consider it as a potential replacement for IAT in most patients (60). However, MEG should not be considered as a replacement for DCS or awake surgery to spare eloquent language areas close to resection borders (60). However, the predictive value of DCS itself is debated and limits its value as a gold standard (21). Ilmberger et al. (61) describe DCS positive sites within the resected lesion as significant risk factor for postoperative language disturbances. However, only 53% of patients with such findings developed a new language-related deficit. Furthermore, postoperative language deficits are often only transient although DCS positive sites have been at least partially resected (62). Additionally, Cervenka et al. (63) reported postoperative deficits in 7 of 11 operated patients which were not anticipated by DCS. This may in part be caused by temporal and financial limitations leading to “incomplete mapping,” as the authors point out.

### Sensorimotor Function

In patients with lesions or epileptogenic zones located at the central region, mapping of sensorimotor cortex is utilized to locate primary somatosensory (S1) and motor (M1) areas (64). While structural landmarks enable the identification of anatomical primary sensorimotor (SM1) cortex, space-occupying lesions may result in considerable displacement and structural alterations. Furthermore, motor areas are especially capable of functional reorganization to neighboring or possibly even remote areas (65). Sensorimotor mapping enables localization of functionally eloquent cortex in the individual patient, and thus the tailoring of resective neurosurgery. Alongside fMRI, DCS and nTMS, MEG has been applied successfully for this indication—for reviews, see (66–68). Studies reporting on EEG–based sensorimotor FCM and its clinical value are however sparse (69, 70) or are evaluated as part of simultaneous EEG/MEG recordings (71, 72).

#### Somatosensory Functional Cortex Mapping

Clinical MEG/EEG mapping of S1 cortex typically utilizes either electrical or mechanical stimulation. The former follows principles of, e.g., median or tibial nerve electrical stimulation as frequently applied in neurological and neurosurgical practice (3, 6, 73–79). Stimulation sites are chosen according to the location of the lesion and the estimated relation to functional cortex. As the duration of the procedure amounts to only a few minutes, multiple stimulation targets can be evaluated, e.g., for comparison with contralateral cortex. Furthermore, stimulation of several sites can be combined into a single measurement run with only slightly extended duration. One study performed in 325 consecutive patients with various brain disorders demonstrated that the success rate of somatosensory mapping based on electrical median and tibial nerve stimulations was significantly lower for the feet than for the hands (95.3% for the hands vs. 76% for the feet) (77). Electrical stimulation however is rather uncomfortable and associated with high-amplitude stimulation artifacts. Stimulation of the face, as well as investigations of children or pain-sensitive patients are therefore usually performed using pneumatic stimulation (80, 81). This procedure utilizes pneumatic stimulation devices with balloon diaphragms, which are moved using pressurized air. Due to the longer latency and higher variability of pressurization, the onset of the evoked activity occurs later and is less sharp. Somatosensory and motor mapping can be combined into a single recording session to limit the overall measurement duration (82). Presurgical somatosensory FCM in patients with brain lesion located close to the central sulcus is, in most instances, used to properly locate the central sulcus and assess likely functional (i.e., motor function) risks associated with resective surgery [see, e.g., (6)]. Still, this approach provides indirect information about the location of motor function, and might therefore be misleading in certain circumstances (e.g., brain lesion inducing substantial anatomical displacement).

#### Motor Cortex Mapping

The spectrum of FCM paradigms and analytical techniques to locate M1 cortex mainly rely on motor evoked fields/potentials (MEF/MEP) and on the suppression of rolandic (alpha and) beta rhythm(s). Additional methods can also be used such as cortico-muscular (CMC) or cortico-kinematic (CKC) coherence.

Motor evoked field/potential or readiness paradigms (73, 77, 83–85) utilize either externally cued or self-paced movements or muscle contractions. Most common are finger tapping and hand closing/opening. Simultaneous recording of EMG (electromyography) enables the exact determination of movement onset. Analysis of the averaged signal then evaluates activity approximately 30–40 ms before movement (60).

Motor activity is accompanied by event-related desynchronization (ERD) or suppression of oscillatory activity in the alpha and beta frequency bands (86). This mu-rhythm, alpha- and beta-band suppression can be localized using, e.g., beamforming (3, 77, 87–89). Paradigms include, e.g., hand grasping (88) and finger extension (3), or ankle flexion/extension (77). Importantly, the beta-band movement-related suppression is organized in a somatotopic manner along the precentral gyrus, while this is less clear for the alpha-band suppression, which has been shown to mainly occur close to the hand region of the postcentral gyrus regardless of the body part moved (90). This explains why beta-band suppression is usually preferred over alpha-band suppression for M1 cortex mapping. The success rate of motor mapping based on movement-related beta-band suppression has been shown to be lower for the feet than for the hands (94.6% for the hands vs. 81.8% for the feet (77).

Coherence approaches (3,88) evaluate the functional coupling of neuronal activity with either muscular activity [as measured by electromyography (EMG)] or movement kinematics. Statistical approaches localize cortical areas of significant coupling with these external reference signals. Stimulation paradigms typically utilize isometric contractions for CMC and recording of (active or passive) movement kinematics with, e.g., accelerometers for CKC (4, 91–93). CMC is considered to reflect mainly the efferent flow of motor commands from M1 cortex to the periphery [for a detailed discussion, see, e.g., (94)] while CKC mainly reflects movement-related somatosensory proprioceptive afferent input to the contralateral SM1 cortex (91, 95).

Of note, MEG and fMRI activations may also be used to support identification of the corticospinal tract (83). Aoyama et al. (96) have combined MEG and tractography for planning of stereotactic irradiation of arteriovenous malformations.

#### Validation and Comparison

MEG-based localization of the SM1 cortex shows high agreement with DCS and fMRI—for a review, see, e.g., (67). Validation based on DCS has been mainly obtained for somatosensory evoked fields (67), to a smaller extent for motor evoked fields (85) or movement-related beta-band suppression (87), and on a few patients for CMC (67). To the best of our knowledge, such validation has not been reported for CKC. Discrepancies between MEG and DCS amount to about 10 mm (81). The comparison of DCS and MEG may however be limited by the spread of the stimulation electrical current, which is largely unknown in the individual case (67), as well as the sensitivity of MEG to sulcal rather than gyral-apical sources. Of note, the intersession reliability of MEG S1 cortex mapping based on electrical median nerve stimulation has been showed to be about 8 mm confidence interval around the estimated location of S1 cortex (79), which is not far from the reported average discrepancy between MEG and DCS.

In comparison to fMRI, MEG shows comparable, and in some patients even superior results (6, 73, 80, 97–102). Mean differences between somatosensory fMRI and MEG results are reported in the range from about 15 mm (73) to 23 mm (70), and for M1 cortex, localizations from 10 mm (73) to 27.9 mm (70). The comparably large variation may be caused by different experimental setups and analysis techniques. Klamer et al. (70), for example, used a distributed source model, whereas, Kober et al. (73) used single equivalent current dipole modeling. In addition, it should be noted that the accuracy of fMRI is also limited by noise, especially in suboptimal recording conditions, which are frequently encountered in clinical practice. High variability may therefore also originate from limited signal-to-noise ratio.

In patients with tumors or vascular lesions in the vicinity of the SM1 cortex, localization of SM1 cortex using fMRI may be difficult due to lesion-induced alterations of regional cerebral blood flow and susceptibility artifacts (103, 104). In these patients, MEG may provide superior results, due to the direct measurement of neuronal activity (105).

MEG also presents an additional key strength over fMRI, which is the ability to investigate in one single MEG session different neurophysiological processes (i.e., evoked magnetic responses, induced magnetic responses, and coupling between peripheral and cortical signals) that can be altered or affected differently by brain lesions or patients' clinical status. Thus, MEG provides the unique opportunity to acquire several MEG “functional localizers” of the SM1 cortex in a reasonable time for the patients (3). “Functional localizer” here refers to a given MEG mapping method to localize the SM1 cortex (see Somatosensory functional cortex mapping and Motor cortex mapping), regardless of the source reconstruction methods used (i.e., equivalent current dipole modeling, minimum norm estimate, spatial filtering approaches). The anatomical convergence of the different MEG functional localizers at the central sulcus has been demonstrated in healthy subjects for hand sensorimotor functional mapping and contributes to the assessment of the confidence level in non-invasive functional mapping results (compared with a uni- or bimodal approach) and to determine the clinical need to undergo further intracranial mapping procedures (3). It also represents a nice way to indirectly validate the localization accuracy of MEG mapping methods not validated by DCS (e.g., CKC) by comparing them with validated methods (e.g., somatosensory evoked fields, motor evoked fields, beta-band suppression). Such approach also increases the yield of MEG in case of failure, inaccurate or atypical localization of one MEG functional localizer or fMRI mapping (3, 67).

Navigated transcranial magnetic stimulation (nTMS) is increasingly applied for presurgical mapping of SM1 cortex (106). A number of studies have shown clinical value for resections of lesions in motor eloquent areas (107–109), as well as a good concordance with DCS (110–113) with, in some cases, smaller distances between nTMS and DCS vs. fMRI and DCS (110). Tarapore et al. (56) evaluated TMS, MEG and DCS for motor mapping in the same population and report distances of 2.13 ± 0.29 mm between TMS and DCS motor sites and 4.71 ± 1.08 mm between MEG and DCS.

Studies evaluating EEG for FCM of SM1 cortex are limited. Klamer et al. (70) compared high-density EEG (hdEEG) and MEG with a similar channel number with fMRI as reference standard. They reported, that, using volume conductor models based on the individual anatomy, source imaging relying on hdEEG may provide localizations that are closer to fMRI than MEG. Mean Euclidean distances were 21.7 mm between EEG and fMRI, and 27.9 mm between MEG and fMRI for motor activity. However, the comparably large deviations of both EEG and MEG suggest that both electromagnetic modalities may be sensitive to different aspects of neural activity than fMRI. This is further supported by deviations of fMRI itself from DCS localizations. Korvenoja et al. (6), for example, reported that fMRI was concordant with intraoperative findings in only 11 of 15 patients. Lascano et al. (69) found good concordance of source imaging relying on hdEEG and fMRI with distances of only 3 to 8 mm. Both, however, deviated from DCS by 13–14 mm. This study further supports the clinical value of EEG especially with a high number of channels. It also highlights the different perspectives of M/EEG, fMRI, and DCS mapping and illustrates the importance of the choice of a gold or, better, reference standard.

### Memory Function

Due to the overlap in declarative memory function and lesions associated with mesial temporal lobe epilepsy (MTLE), memory impairment is common in this group of patients. Verbal memory decline can be observed in 30–85% of patients who undergo left temporal resection, whereas non-verbal memory deterioration after right (- or left) temporal resection affects 30–50% (51, 114–116).

IAT is considered the gold standard for the assessment of declarative memory function. Impaired memory performance is usually found in about 20–30 % of cases injected ipsilateral to the seizure onset zone and in 60–80% after contralateral injection (117–119). Based on these results, several authors consider IAT results as a prognostic tool to predict postsurgical declarative memory, although results on the predictive value of IAT are contradictory and controversial (120–123), and memory results on repeated IAT are much less robust than those of verbal language testing (15).

In addition to the methodological drawbacks of IAT, there is no guarantee that amobarbital can be sufficiently delivered to the targeted hippocampal formation (124, 125).

fMRI, MEG, PET, and TMS, as well as several combined/integrated methods are non-invasive alternatives evaluated for declarative memory functional mapping (126).

In this context, fMRI is by far the most popular and widely used solution. For a comprehensive review, see e.g., (122).

To the best of our knowledge, MEG (or EEG) are not in (routine or validated) clinical use for declarative memory FCM. The most important obstacle might be the detection of hippocampal activation due to the limited sensitivity of MEG to deep sources—a problem that has been addressed, leading to the implementation of research protocols including the evaluation of deep structures via MEG (127–130). Clinical research in this area remains sparse. Maestú et al. (131) investigated verbal episodic memory in 9 patients with left MTLE in comparison to 9 healthy controls. MEG showed a left-hemisphere-dominant activation pattern in healthy controls, whereas patients' activation patterns showed mainly right-hemispheric dominance. Three patients underwent left anterior temporal lobectomy. They showed no significant postoperative memory loss and Engel class 1A/B outcome. These data suggest, that MEG has the potential to be used for memory FCM, but further studies are clearly needed.

#### Validation and Comparison

Even though it is considered the gold standard, the role of IAT in the prediction of postoperative declarative memory outcome remains controversial. Rathore et al. (123) reported memory outcome data on 116 patients after left anterior temporal lobe resection. Approximately one third of patients had “failed” IAT, meaning that test results indicated ipsilateral memory representation. After resection (operation was performed regardless of IAT-results), no difference was found between the group who failed and those who passed the IAT.

fMRI results show good concordance with IAT, which is also the basis for most validation studies. Throughout the literature, there is a great heterogeneity regarding the experimental paradigms used, but verbal memory tasks show the most consistent and clinically useful results (132).

To the best of our knowledge no validated method of declarative memory functional mapping relying on MEG or EEG exists.

### Visual Cortex

Damage to the visual cortex or optic radiations may result in partial or complete anopia, whereas congenital defects or lesions might result in functional reorganization (133, 134). Presurgical FCM provides localization information of potentially displaced primary visual (V1) cortex either using EEG, MEG, or fMRI and thus helps to avoid such damage. Due to the fact that partial anopia is rather accepted by doctors and patients in certain circumstances, visual FCM is of limited clinical value. Therefore, functional mapping of V1 cortex is rarely applied in comparison to, e.g., FCM of verbal language or SM1 areas. Correspondingly, literature on application in clinical settings is sparse, in comparison to studies focusing on basic neuroscientific research of the visual system.

Early components of visual evoked activity in EEG and MEG (visual evoked potentials—VEP and visual evoked fields—VEF) localize to V1 cortex. Paradigms apply pattern reversal stimuli, such as checkerboards, presented to a hemifield or a single quadrant (135–137). Sources of early evoked activity occurring approximately 100 ms after stimulus onset (135, 138), i.e., pattern reversal, can be modeled using single equivalent current dipolar models (135). Presence of a lesion may result in prolonged latencies (139). Robustness of the method is critically dependent on stimulation quality, which is substantially influenced by the projection equipment and the variability of stimulus onset. Similar to approaches in language and motor systems (45, 83), MEG activations have been combined with tractography to support identification of V1 and the optic radiation (140).

#### Validation and Comparison

EEG-based visual evoked potentials are used for intraoperative neurophysiological monitoring, which evaluates response latencies and amplitudes rather than localization (141). Studies on presurgical EEG-based FCM of V1 cortex are lacking.

DCS, e.g., during invasive Video-EEG monitoring for presurgical evaluation of refractory focal epilepsy, provides high accuracy and selectivity for different aspects of the visual system (142). A comparison of MEG mapping with DCS has not been performed. In principle, TMS can stimulate and thus localize V1 cortex (143), however, clinical evidence is lacking. Consequently, there are no data available relating M/EEG to TMS.

fMRI has shown the ability to accurately localize V1 cortex and differentiate correlates of different stimulus aspects, such as visual field eccentricity and angular position by sophisticated paradigm design (144). Localization accuracy has been evaluated in comparison to DCS in a handful of studies (145, 146), which nevertheless show very high concordance rates. Comparisons between EEG and MEG V1 cortex FCM are not available.

### Auditory Cortex

Auditory mapping is clinically applied in patients with lesions or structural alterations within or near Heschl's gyrus (147). Lesional growth may lead to displacement and also reorganization of primary auditory areas. Identification by using anatomical landmarks alone may be challenging in few cases. Cortical deafness as a result of a lesion or surgical procedure is, however, a rare complication due to the redundant bilateral representation (148). This explains why auditory FCM is actually of limited clinical usefulness. Latencies and amplitudes of, e.g., the N100 response or its magnetic counterpart M100 (or N100 m) may be changed in patients with autism (149, 150), dyslexia (151), corticobasal degeneration (152), ischemic lesions (153), and tumors (147).

Auditory stimulation in EEG/MEG recordings typically use monaural presentation of brief sine or click tones with white noise masking of the contralateral ear (60). Averages of 200–500 trials are then analyzed regarding amplitudes and latencies. Localization analysis usually focuses on the N/M100 response and relies on equivalent current dipole modeling. Early brainstem auditory evoked potentials are not well-recorded with MEG (60), but are detectable when using a considerably larger number of averages (154).

Uni- and bilateral auditory stimulation results in bilateral activation of auditory cortices, reflected by bilateral dipolar generators. In EEG, this leads to merging potentials with a maximum negativity over the vertex and positivity over the basal temporal lobes. Due to the different sensitivity and rotated orientation of magnetic fields, the two components are easier to separate in MEG. Furthermore, generators of auditory activity have a predominantly tangential orientation, which improves SNR in MEG (155).

#### Validation and Comparison

Early studies have shown the accuracy of MEG (156, 157) and EEG (158) to localize auditory evoked activity (159). Scarff et al. (160) conducted EEG-fMRI measurements of auditory evoked activity. They reported good concordance of both methods in the horizontal plane. However, EEG dipoles localized more cranially than fMRI. Increasing the number of electrodes from 64 to 128 improved the concordance. Shahin et al. (161) evaluated auditory activity with simultaneous recordings of MEG and EEG and reported a good concordance regarding localization, although source amplitudes differed depending on the orientation of generators. Between-subject-variability of localizations was lower in MEG compared to EEG. Studies comparing accuracy of MEG, EEG and DCS or nTMS for identification of the auditory cortex in a clinical context are not available.

### New Approaches

The evaluation of functional connectivity has gained considerable interest in the last years, partially due to methodological and technical advances. Going beyond the absolute activation and focusing on functional integration patterns between brain areas has opened new opportunities for FCM. These new approaches enable identification of functional networks, rather than task-based individual activated areas. The main hypothesis assumes that this provides a more complete and realistic view of human brain function. While most studies currently focus on physiological activity in healthy subjects, there are promising findings with potential for novel clinical application (162). For example, Doesburg et al. (163) showed functional connectivity and cross-frequency modulation in the gamma and theta frequency bands during a verb generation paradigm in expressive language networks. These findings did not necessarily coincide with the localization of, e.g., task-based gamma power modulations. Although they observed this functional connectivity structure in left- and right-sided regions, cross-frequency modulations were more pronounced in the left frontal cortex and particularly in the inferior frontal gyrus. Such features of neuronal communication may therefore represent candidates for potentially more specific FCM of, e.g., verbal language-related brain areas.

Evaluation of functional connectivity patterns in the resting brain (i.e., in the absence of any explicit task) potentially also provides relevant information for localization of functionally eloquent cortices in fMRI (164) and MEG (165). Martino et al. (164) evaluated such resting state functional connectivity (rsFC) in the vicinity of tumors and compared the results with DCS. Decreased rsFC in the tumor vicinity was associated with absence of eloquent cortex (via DCS) in all cases, while increased rsFC indicated the presence of eloquent cortex (via DCS) in 64% of patients. Tarapore et al. (165) utilized the rsFC method in MEG to predict postoperative functional deficits in patients undergoing glioma surgery. They showed that patients with increased rsFC in the tumor area presented with new neurological deficits in 25% of cases at 6 months after surgery vs. 0% in patients with decreased rsFC. These findings are corroborated by similar results using resting state fMRI data (166). The main limitation of such approaches is currently the limited availability of clinical studies. In addition, analysis methodology is complex and diverse, with little standardization at this point. Furthermore, the specific functional significance of individual resting state and connectivity measures remains largely unclear (166) and requires further investigation.

Machine learning algorithms are transforming multiple medical fields. Clinical applications are already in use in various domains—predominantly in image processing, classification and segmentation (167). Roland et al. (168) used a machine learning approach on resting state fMRI for sensorimotor cortex mapping and compared results with DCS in 16 pediatric patients with epilepsy. The authors report comparable functional localization between the two methods.

In the future, machine learning might find further application in FCM, e.g., based on methods and findings in the field of brain-computer interfaces (169). However, specific analysis protocols, thorough evaluation of the applied algorithms and clinical validation are lacking at this time.

Finally, studies demonstrated that FCM based on the investigation of event related enhancement of high gamma activity as recorded by invasive recordings or electrocorticography during awake craniotomy might be of high interest for the mapping of verbal language and motor eloquent cortex (170–173). Still, this approach is mainly used in some specialized centers and further validation studies are needed.

## Discussion

For any validation of FCM methods, the choice of the reference standard used to validate the methods is crucial. DCS either intraoperatively or during invasive monitoring with subdural or depth electrodes is considered as one gold standard. However, data on current flow from the electrodes through the cortex is sparse in addition to how such currents interact with the neuronal architecture beyond single cell responses. In addition, different methods might be sensitive to different aspects of neural activity. Lascano et al. (69) described a systematic difference between hdEEG and fMRI regarding S1 cortex localization, with fMRI showing more lateral activation. The authors argued that this discrepancy may be caused by EEG detecting early activity in Brodmann area 3b, while fMRI would reflect more integrative processes in the more lateral Brodmann area 1.

In addition, the predictive value of DCS itself may be limited, as illustrated by Ilmberger et al (61). The authors report on the outcome of intraoperative mapping of language functions in 149 tumor patients. One of the main risk factors for early postoperative disturbances was a DCS language-positive site within the tumor. However, only 53% of these patients developed a new postoperative deficit. Furthermore, 7 months after surgery, only pre- or postoperative aphasia, and increased age were associated with persisting deficits. Positive stimulation sites within the resected tumor did no longer show a significant influence. While subtle deficits of language function may not have been considered, the predictive value of DCS was therefore limited to early postoperative language function in a portion of the patients. It ultimately did not affect the outcome after this initial phase. This overestimation of functional involvement, as also found in the non-invasive alternatives, does not necessarily contradict clinical utility. However, it imposes limits on the validity of DCS as a “ground-truth” gold standard, potentially also since it does not and potentially cannot take postoperative reorganization phenomena into account.

In a prospective clinical study by Hermann et al. (46), resection of presumed verbal language eloquent cortex in the superior temporal gyrus was compared with sparing these areas. Postoperative outcomes showed no significant difference in visual confrontation naming. Since intraoperative DCS was not performed, it can only be assumed but is not proven, that language eloquent cortex was resected. As patients underwent classical anterior temporal lobe resections it should be pointed out, that results arguably might have been different, if more extensive resective approaches had been applied. Furthermore, evaluation of subtle verbal language deficits is challenging, especially as postoperative reorganization may mask these.

Tate et al. (174) identified areas in the non-dominant hemisphere, the stimulation of which led to speech arrest. However, only patients with low grade glioma were evaluated, implying that such reorganization may have led to a shift of verbal language-related areas.

Especially in patients with atypical hemispheric dominance, all non-invasive methods show limitations. Bauer et al. (175) published a meta-analysis of fMRI vs. IAT for verbal language lateralization. They reported a concordance rate of 91% in patients with typical language lateralization. However, in patients with atypical lateralization, concordance rate dropped to only 51%. Similarly, while MEG-based verbal language lateralization was concordant with IAT in 32 of 35 patients in a study by Tanaka et al. (176), the remaining were patients with bilateral language representation. In addition, Picht et al. (112) reported overestimation of areas involved in verbal language processing by TMS, underlining that this issue does not seem to be a problem of a single modality, e.g., due to limitation of data quality, etc.

In light of these limitations, non-invasive localization methods that seek to go beyond mere lateralization can provide only a conservative estimate of essential functional cortex. If such areas are spared by the neurosurgical procedure, functional deficit can thus probably be avoided in most cases. However, on the other hand, a portion of these regions could potentially be resected without any functional deterioration, e.g., to achieve gross total resection in tumor surgery or to completely remove the epileptogenic zone. In the context of epilepsy surgery, this “conservative” perspective on functionally eloquent cortex may be warranted, especially as quality of life is the central goal. However, tumor surgery requires more aggressive strategies, which would benefit from increased specificity. Focusing on patients undergoing tumor surgery might therefore help to better determine if this conservative approach is indeed mandatory in all situations.

The current challenge is to identify markers, which provide a more accurate and robust estimation of this essential, necessary cortex.

In general, studies on the direct comparison of different FCM methods in the same patient population are sparse. Most publications focus on the diagnostic accuracy of a single method vs. a mostly invasive reference standard, i.e., DCS or IAT, etc. When postsurgical outcomes are considered, follow-ups are frequently short. Only limited data is available relating presurgical FCM to long-term outcomes and functional reorganization. Results on clinical M/EEG FCM of the auditory or visual system are generally sparse, potentially due to the limited clinical relevance. A main issue for validation of not only M/EEG-based FCM, but also the prognostic value of fMRI, TMS, and even DCS is the very limited availability of prospective, randomized clinical studies.

While EEG and MEG register similar activity with differences in sensitivity, EEG is rarely applied for FCM in a clinical context. This is in stark contrast to the wide utilization of EEG in clinical routine for diagnosis and in neurocognitive studies (177), although these latter ones frequently focus on group sensor-level results rather than specific brain localizations at the individual level. HdEEG is a promising alternative to fMRI and MEG for FCM in a clinical setting though it requires more studies focusing on this application.

Both MEG and EEG are sparsely used for FCM in tumor patients in comparison to, e.g., fMRI, while most clinical MEG centers perform functional mapping in the context of epilepsy surgery (178, 179). Reasons for the sparse application may be limited availability of reimbursement in many countries and the subsequent constrained access to MEG, but also HdEEG. Furthermore, a wide spectrum of methodological approaches without a clear gold-standard procedure complicates implementation and application in clinical routine, while technical challenges, e.g., to integrate results into neuronavigation, have been solved (81, 180, 181). Further development of existing clinical practice guidelines (60, 182) as well as comparative and prospective studies would certainly impact practical application.

In conclusion, electromagnetic source imaging provides additional information for functional mapping with reasonable spatial resolution, exquisite temporal resolution and direct information about neural activity. Due to their non-invasive nature, these methods can be applied early in the presurgical workup and can be utilized to optimize the application of invasive means, such as DCS. Further evaluation is needed to investigate their respective clinical added-value.

## Author Contributions

All authors listed have made a substantial, direct and intellectual contribution to the work, and approved it for publication.

### Conflict of Interest Statement

The authors declare that the research was conducted in the absence of any commercial or financial relationships that could be construed as a potential conflict of interest.
